# Prevalence and mortality risk of low skeletal muscle mass in critically ill patients: an updated systematic review and meta-analysis

**DOI:** 10.3389/fnut.2023.1117558

**Published:** 2023-05-12

**Authors:** Hui Yang, Xi-Xi Wan, Hui Ma, Zhen Li, Li Weng, Ying Xia, Xiao-Ming Zhang

**Affiliations:** ^1^Department of Nursing, Chinese Academy of Medical Sciences-Peking Union Medical College Hospital, Beijing, China; ^2^Department of Medical Intensive Care Unit, Chinese Academy of Medical Sciences-Peking Union Medical College Hospital, Beijing, China; ^3^Department of Urology, Chinese Academy of Medical Sciences-Peking Union Medical College Hospital, Beijing, China

**Keywords:** prevalence, mortality, low skeletal muscle mass, critically ill patients, systematic review and meta-analysis

## Abstract

**Background:**

Patients with critical illness often develop low skeletal muscle mass (LSMM) for multiple reasons. Numerous studies have explored the association between LSMM and mortality. The prevalence of LSMM and its association with mortality are unclear. This systematic review and meta-analysis was performed to examine the prevalence and mortality risk of LSMM among critically ill patients.

**Methods:**

Three internet databases (Embase, PubMed, and Web of Science) were searched by two independent investigators to identify relevant studies. A random-effects model was used to pool the prevalence of LSMM and its association with mortality. The GRADE assessment tool was used to assess the overall quality of evidence.

**Results:**

In total, 1,582 records were initially identified in our search, and 38 studies involving 6,891 patients were included in the final quantitative analysis. The pooled prevalence of LSMM was 51.0% [95% confidence interval (CI), 44.5–57.5%]. The subgroup analysis showed that the prevalence of LSMM in patients with and without mechanical ventilation was 53.4% (95% CI, 43.2–63.6%) and 48.9% (95% CI, 39.7–58.1%), respectively (*P*-value for difference = 0.44). The pooled results showed that critically ill patients with LSMM had a higher risk of mortality than those without LSMM, with a pooled odds ratio of 2.35 (95% CI, 1.91–2.89). The subgroup analysis based on the muscle mass assessment tool showed that critically ill patients with LSMM had a higher risk of mortality than those with normal skeletal muscle mass regardless of the different assessment tools used. In addition, the association between LSMM and mortality was statistically significant, independent of the different types of mortality.

**Conclusion:**

Our study revealed that critically ill patients had a high prevalence of LSMM and that critically ill patients with LSMM had a higher risk of mortality than those without LSMM. However, large-scale and high-quality prospective cohort studies, especially those based on muscle ultrasound, are required to validate these findings.

**Systematic review registration:**

http://www.crd.york.ac.uk/PROSPERO/, identifier: CRD42022379200.

## 1. Introduction

The intensive care unit (ICU), created in 1953, has become an integral part of the health care system worldwide for patients with critical illness (1). The survival rates of patients with critical illness have improved because of major progress in medical technology, greater understanding of disease pathophysiology, and use of multidisciplinary approaches to care (2). However, improving the prognosis of critically ill patients remains an important issue for critical care departments. Numerous risk factors are closely related to the mortality of critically ill patients, such as undernutrition (3), multiple organ failure (4), advanced age (5), sepsis (6), muscle wasting, frailty (7), and other factors. Among these factors, muscle wasting has drawn increasing attention from intensive care physicians.

Muscle wasting, also termed myopenia, is defined as wasting or thinning of muscle mass (8). Muscle wasting is assessed using a semiautomatic method of measuring the amount of muscle area on a computed tomography (CT) scan using predefined Hounsfield units (9). The skeletal muscle index is then computed by dividing the estimated muscle area by the body height. A reasonable threshold for prediction of low skeletal muscle mass (LSMM) has been suggested to be a skeletal muscle index in the fifth age-matched percentile (10). Previous studies have confirmed that older people with muscle wasting have a high risk of falls (11), mortality (12), fractures (13), and functional decline (14), which lead to adverse impacts on the economy and society as well as increased healthcare expenditures (15). Therefore, management of LSMM has become an important issue. Numerous methods have been adopted to assess muscle mass, including magnetic resonance imaging, CT, ultrasound, dual-energy X-ray, bioelectrical impedance, and anthropometric methods (16). The most commonly used method in critically ill patients is CT.

In recent decades, LSMM has become a focus of research in critical care. Critically ill patients can easily develop LSMM secondary to malnutrition, inactivity, and inflammatory reactions (17). Several studies have explored the association between LSMM and adverse outcomes among critically ill patients (18–20). These studies showed that the presence of LSMM based on CT scans was associated with a high risk of all-cause death among critically ill patients (18, 21) and that measurement of the total psoas muscle area can improve the prediction of mortality (22). In addition, many studies have shown that the prevalence of LSMM among critically ill people is higher than that among older people (23, 24). A recent systematic review revealed an LSMM prevalence of 50.9% (25). However, this review consisted of only 9 studies involving 1,563 patients, and the studies used only CT to assess muscle mass. Some studies have detected LSMM by newer technologies such as ultrasound (26, 27). Most importantly, the above-mentioned systematic review did not perform a subgroup analysis. Moreover, many new articles have explored the impact of LSMM on mortality (18, 19, 21, 28–35). Therefore, we considered it very important to perform an updated systematic review to summarize the prevalence and mortality risk of LSMM in critically ill patients. The aim of our study was to systematically summarize the prevalence of muscle wasting among critically ill patients and identify whether critical illness with LSMM can increase the risk of mortality.

## 2. Methods

### 2.1. Search strategy

This systematic review is reported in accordance with the PRISMA guidelines and was preregistered in the PROSPERO database (CRD42022379200). Two authors searched for relevant articles in three internet databases (PubMed, Embase, and Web of Science) from database inception to 1 September 2022. We used the following keywords and Medical Subject Headings (MeSH) terms to identify relevant studies: “muscle mass” or “muscle wasting” or “low skeletal muscle” and (“mortality” or “death” or “survival”) and “critically ill patient”. The detailed search strategy is shown in Supplementary File 1.

### 2.2. Inclusion and exclusion criteria

The patients, intervention, comparison, outcomes, and study design (PICOS) principle was adopted to confirm study eligibility. The inclusion criteria were as follows: (1) the patients involved in the study were critically ill (i.e., adult patients treated in the ICU); (2) as the exposure, LSMM was definitively diagnosed based on CT scans, anthropometric methods, and ultrasound; (3) the article presented the prevalence of LSMM, or the prevalence could be calculated using the data available within the article; and (4) the study design was observational (cohort study or cross-sectional study). Reviews, case reports, comments, correspondence articles, letters, and abstracts were excluded because complete quality assessment of such reports could not be performed.

### 2.3. Study selection and data extraction

Two authors independently formulated the search strategy and screened the articles. First, the results of the relevant studies from the three databases were imported into EndNote X9 software, and duplicates were deleted. Next, the authors screened the title and abstract based on the PICOS principle, checked the abstract for potential relevance, and screened the full text. The final studies were confirmed after careful review of the full text. During this process, disagreements were resolved by discussion with a third reviewer. Two authors also independently extracted the data based on standardized forms consisting of author, year, country, main diagnosis, age, sample size, prevalence of muscle wasting, number of female/male participants, prevalence of muscle wasting by sex, muscle wasting assessment tool used, and effect size of the association between LSMM and mortality.

### 2.4. Assessment of study quality

We used the Newcastle–Ottawa Scale to assess the quality and methodology of the included studies. The assessing item including selection, comparability, and outcome. To minimize the potential for bias, we had two reviewers independently evaluate each included study using the Newcastle-Ottawa Scale, and we resolved any discrepancies through discussion and consensus. The total score of the included studies ranged from 0 to 9 points, and the quality of the study was defined as poor, moderate, or high with corresponding scores of 0–4, 5–6, and 7–9 points, respectively.

### 2.5. The quality of evidence

We used GRADE tool to assess the overall quality of the evidence. This tool consisted of five items including risk of bias, inconsistency, indirectness, imprecision and publication bias.

### 2.6. Outcome measures

The primary outcome of this systematic review was the prevalence of LSMM, and the secondary outcome was all-cause mortality.

### 2.7. Statistical analysis

All statistical analyses were performed with Stata Version 14 (StataCorp, College Station, TX, USA). Metaprop, a Stata command, was used to pool the prevalence of muscle wasting from each included study, and the metan command was used to combine the results regarding the association between LSMM and mortality risk of all studies. A random-effects model was used because of the high heterogeneity (*I*^2^ > 50%) across studies caused by differences in countries, definitions, sample sizes, and reasons for ICU admission. In addition, to detect the original cause of heterogeneity, different subgroup analyses based on country, sex, sample size (<100 vs. ≥100), age group, main diagnosis for ICU admission, mechanical ventilation, and type of outcome were performed if there were more than two studies within each stratum. Finally, a sensitivity analysis and test of publication bias were performed.

## 3. Results

### 3.1. Study selection

**Figure 1:**
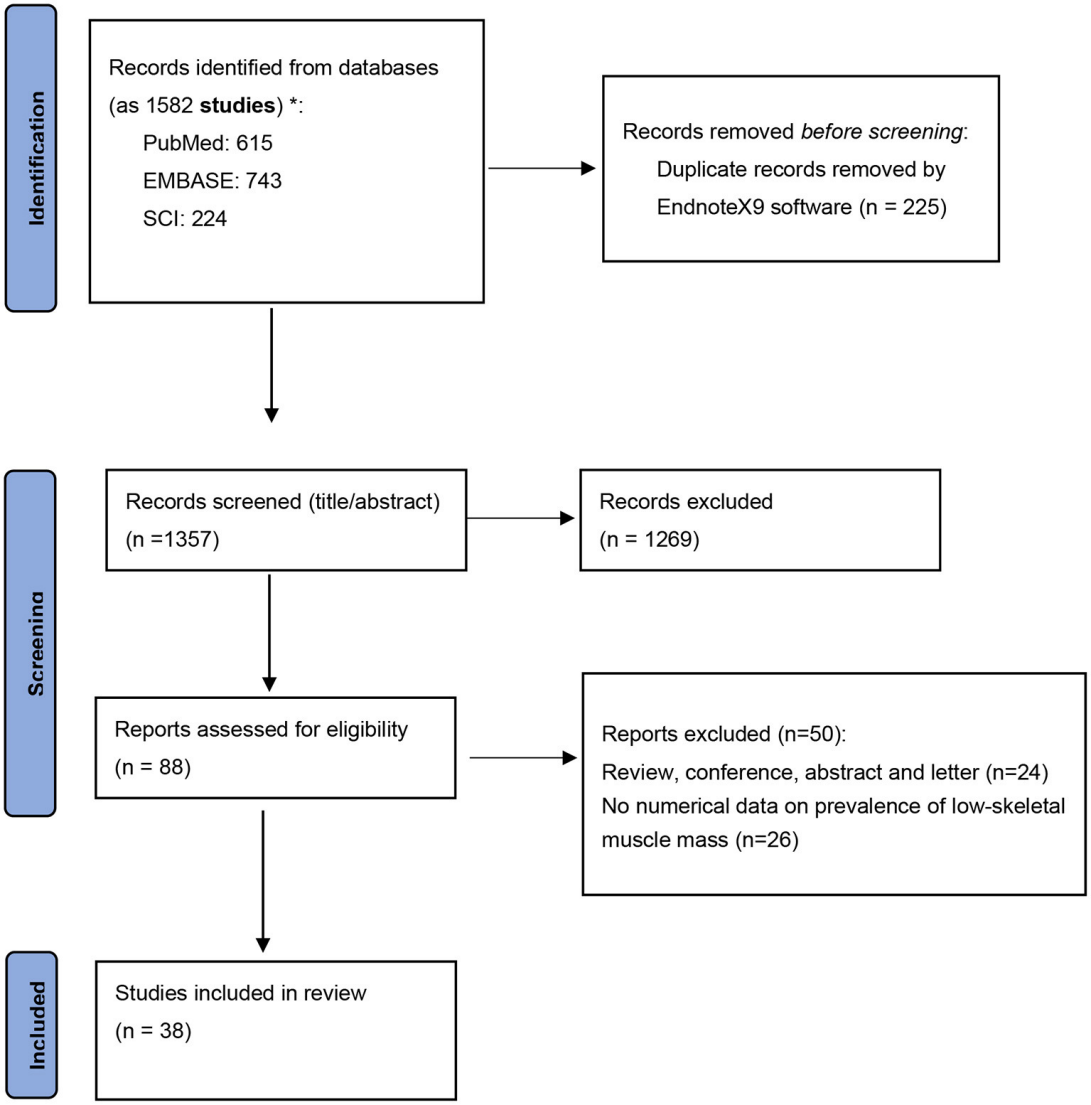
Flow diagram of studies selection.

In total, 1,582 records were identified from 3 databases (PubMed, *n* = 615; Embase, *n* = 743; and Web of Science, *n* = 224). After deleting duplicates, 1,357 studies remained to be screened. Two authors deleted 1,269 studies after checking the title and abstract, resulting in 88 studies for full-text review. Of these, 38 studies were included in the final quantitative analysis based on the inclusion and exclusion criteria (18–21, 24, 26–58). The main reasons for exclusion were an ineligible study design and irrelevant studies exploring the association between LSMM and other clinical outcomes (Figure 1).

### 3.2. Characteristics of included studies

Thirty-eight studies involving 6,891 participants met the eligibility criteria. The participants' mean or median age ranged from 41.4 to 79 years. Most of the studies were conducted in the United States (*n* = 9), followed by Korea (*n* = 7) and Japan (*n* = 6). Four studies were performed in the Netherlands, three in China, and two in Italy. Only one study each was conducted in Brazil, India, Germany, and Malaysia. The main diagnosis among the participants in the included studies was sepsis (14 studies), trauma (7 studies), surgical diseases (3 studies), and COVID-19 (1 study). Thirteen studies involved patients with mixed diagnoses collectively referred to as “critical illness”. The highest prevalence of LSMM was 90%, and the lowest prevalence was 25%. The largest sample size of the included studies was 905 (33), and the smallest sample size was 37 (31). A total of 89.4% of studies used CT scans to assess muscle mass, whereas only two studies used ultrasonography (26, 27) and two used anthropometric methods (19, 50). Thirty studies explored the association between LSMM and mortality among critically ill patients (Table 1). Thirteen studies considered the in-hospital mortality as the main outcome and six studies reported 30-day mortality, followed 6 studies for 1-year mortality. Supplementary Table 1 displayed the time for assessing muscle mass.

**Table 1:** Characteristics of the included studies.

**References**	**Country**	**Mechanical ventilation**	**Design**	**Age/mean (SD)/median**	**Main diagnosis**	**Total sample size**	**Prevalence of LSMM**	**Muscle mass assessment tool**	**Type of outcomes**
Akahoshi et al. (36)	Japan	Yes	Retrospective study	49.95 ± 16.3	Trauma	84	0.30	CT	30-day mortality
Baggerman et al. (37)	Netherlands	No	Retrospective cohort study	66.0 ± 13.6	Sepsis	155	0.31	CT	In-hospital mortality
Bareto (38)	USA	No	Retrospective cohort study	63.4 ± 16.3	Sepsis	81	0.70	CT	NA
Cho et al. (39)	Korea	Yes	Retrospective cohort study	≥18	Critically ill	127	0.37	CT	1-year mortality
Cox et al. (28)	USA	No	Prospective cohort study	53 ± 14	Sepsis	47	0.49	CT	30-day mortality
Damanti et al. (40)	Italy	Yes	Cross-sectional	59.3 ± 11.91	COVID-19	81	0.65	CT	NA
Ebbeling et al. (42)	USA	Yes	Prospective study	74 ± 3.17	Trauma	180	0.50	CT	In-hospital mortality
de Hoogt et al. (41)	Netherlands	No	Retrospective cohort study	None	Critically ill	139	0.32	CT	In-hospital mortality
Hwang et al. (43)	USA	No	Retrospective cohort study	63.7 ± 16.4	Critically ill	230	0.32	CT	In-hospital mortality
Ji et al. (44)	China	Yes	Retrospective study	68.75 ± 4.17	Sepsis	236	0.48	CT	30-day mortality
Joyce et al. (24)	Australia	Yes	Retrospective study	63.7 ± 16.4	Sepsis	279	0.68	CT	30-day mortality
Ju et al. (45)	Korea	Yes	Prospective study	64.3 ± 11.2	Critically ill	125	0.90	CT	NA
Kaplan et al. (46)	USA	No	Retrospective cohort study	None	Trauma	450		CT	1-year mortality
Khan et al. (29)	India	Yes	Prospective study	48.37 ± 11.29	Critically ill	111	0.68	CT	ICU-mortality
Kim et al. (30)	Korea	No	Case-control	≥18	Sepsis	516	0.82	CT	1-year mortality
Koga et al. (47)	Japan	No	Retrospective study	≥18	Sepsis	191	0.48	CT	In-hospital mortality
Kou et al. (48)	China	Yes	Retrospective study	68.75 ± 4.17	Surgery	96	0.31	CT	In-hospital mortality
Looijaard et al. (49)	Netherlands	No	Prospective study	59 ± 17	Critically ill	110	0.47	CT	NA
Malle et al. (31)	Australia	No	Retrospective study	58.8 ± 17.3	Critically ill	37	0.49	CT	6-month Mortality
Moisey et al. (51)	USA	Yes	Retrospective cohort study	79 ± 2.7	Trauma	149	0.71	CT	In-hospital mortality
Moon et al. (32)	Korea	Yes	Retrospective study	78 ± 1.33	Sepsis	190	0.51	CT	In-hospital mortality
Loosen et al. (58)	Germany	Yes	Retrospective cohort study	60 (21–88)	Critically ill	155	NA	CT	1-year mortality
Lucidi et al. (50)	Italy	No	Retrospective study	49.7 ± 16	Sepsis	74	0.43	Anthropometric	In-hospital mortality
Mueller et al. (27)	USA	No	Prospective study	61.9 ± 15.8	Critically ill	102	0.43	Ultrasound	In-hospital mortality
Ng et al. (52)	Malaysia	Yes	Retrospective study	54.4 ± 17.8	Critically ill	228	0.50	CT	In-hospital mortality
Oh et al. (33)	Korea	No	Retrospective study	65.7 ± 15.0	Sepsis	905	0.45	CT	1-years mortality
Okada et al. (34)	Japan	No	Retrospective study	76 (64–84)	Sepsis	255	0.33	CT	90-day mortality
Proksch et al. (35)	USA	No	Prospective cohort study	70	Trauma	76	0.50	CT	6-month mortality
Seo et al. (53)	Korea	No	Retrospective study	65.0 (58.0–72.0)	Sepsis	175	0.86	CT	30-day mortality
Sheean et al. (54)	USA	Yes	Cross-sectional	59.2 ± 15.6	Sepsis	56	0.61	CT	NA
Shibahashi et al. (55)	Japan	No	Retrospective cohort study	75 (68–82)	Sepsis	150	0.55	CT	In-hospital mortality
Shibahashi et al. (56)	Japan	No	Retrospective cohort study	>60	Trauma	74	0.54	CT	NA
Toledo et al. (20)	Brazil	No	Retrospective cohort study	61.6 ± 13.5	Critically ill	99	0.38	CT	30-day mortality
Vongchaiudomchoke et al. (19)	Thailand	Yes	Prospective study	75.0 ± 7.6	Surgery	120	0.33	Anthropometric	120-day mortality
Weijs et al. (57)	Netherlands	Yes	Retrospective study	59.5 ± 17.8	Critically ill	240	0.63	CT	In-hospital mortality
Woo et al. (18)	Korea	Yes	Retrospective study	66.4 ± 14.5	Surgery	45	0.67	CT	NA
Xi et al. (21)	China	Yes	Retrospective study	41.4 ± 15.9	Trauma	451	0.25	CT	NA
Yanagi et al. (26)	Japan	No	Prospective cohort study	70 (60–76)	Critically ill	72	0.36	Ultrasound	1-year mortality

CT, computed tomography scan; NA, not available.

### 3.3. The diagnosis criteria and cut-off points for LSMM of each study

Based on the information provided, the diagnosis criteria and cut-off points for LSMM among the included studies were summarized in Supplementary Table 2. For CT-scans, the majority of studies used skeletal muscle mass index (SMI) to define LSMM. Some studies used skeletal muscle area (SMA), and only two studies adopted total psoas area (TPA). In addition, ultrasonography was used in some studies to assess the Femoris Muscle for confirming LSMM. Whereas, the cut-off value for confirming LSMM were varied across these studies.

### 3.4. Meta-analysis of prevalence of LSMM in critically ill patients

**Figure 2:**
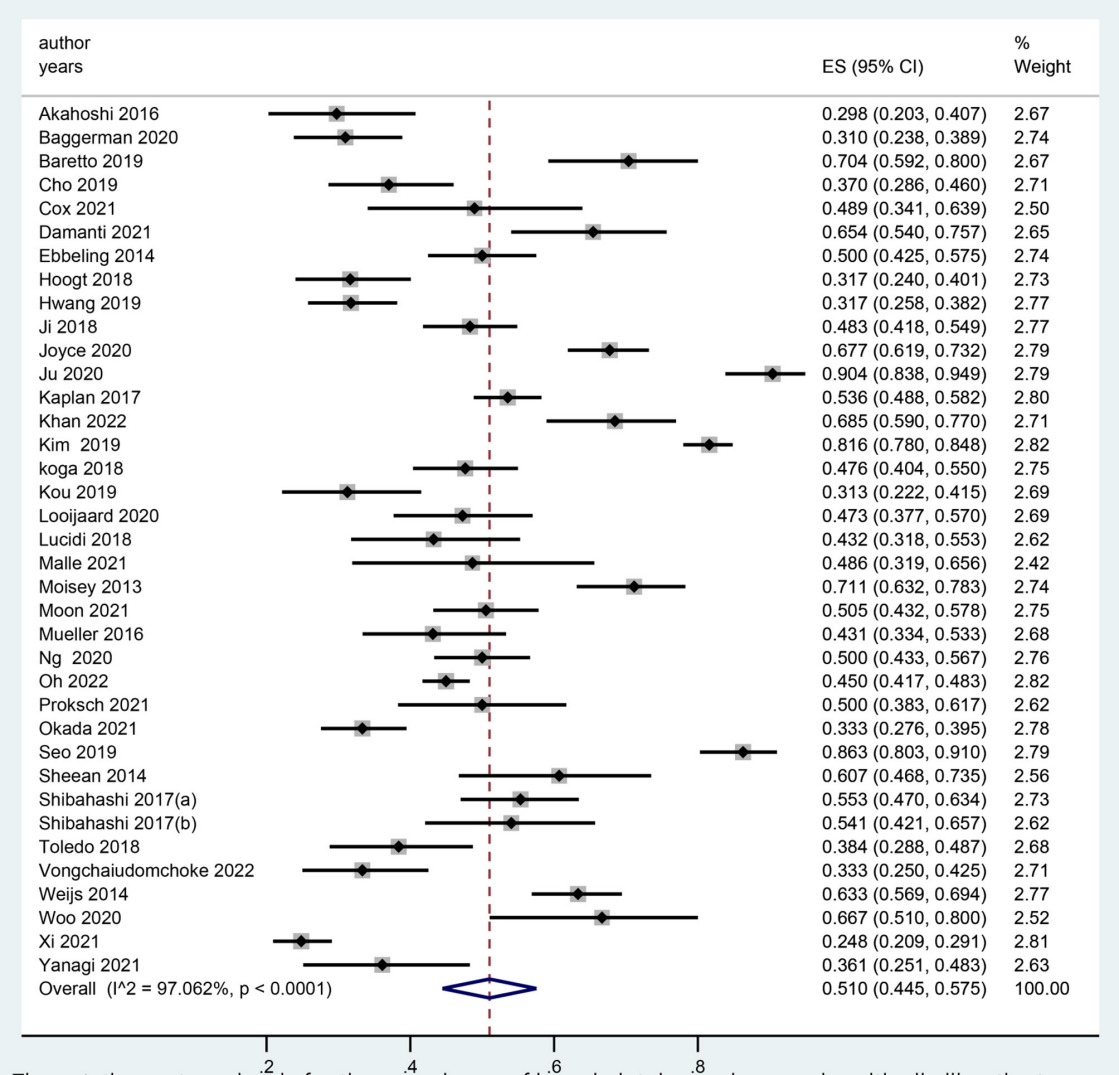
The meta-anlaysis for the prevalence of low skeletal muscle mass in critically ill patients.

In total, 37 studies reported the prevalence of LSMM among critically ill patients (24, 27–33, 36–52). The prevalence ranged from 25 to 90%, and the pooled prevalence of LSMM was 51.0% [95% confidence interval (CI), 44.5–57.5%] (Figure 2).

### 3.5. Subgroup analyses of pooled prevalence by different variables

#### 3.5.1. Subgroup analysis by region

The results of the subgroup analysis of the pooled prevalence of LSMM based on region showed that the prevalence of LSMM was 51.1% (95% CI, 40.6–61.6%) among Asians, 46.9% (33.9–60.0%) among Europeans, 51.7% (43.2–60.2%) among Americans, and 65.8% (60.6–70.9%) among Oceanians (Table 2).

**Table 2:** Subgroup analyses for the meta-analysis of prevalence of LSMM among critically ill patients.

**Variables**	**Number of studies**	**Prevalence**	**95%CI**	***I* ^2^**	***P*-value for difference**
**Country**					0.72
Asian	19	51.1%	40.6–61.6%	98.21%	
Europeans	6	46.9%	33.9–60.0%	93.23%	
Americas	10	51.7%	43.2–60.2%	90.64%	
Oceanias	2	65.8%	60.6–70.9%	97.06%	
**MV**					0.44
Yes	17	53.4%	43.2–63.6%	97.14%	
No	20	48.9%	39.7–58.1%	97.28%	
**Gender**					0.51
Male	25	48.8%	40.0–57.6%	96.75%	
Female	25	45.5%	37.9–53.2%	92.80%	
**Diagnose**					0.65
Trauma	7	47.6%	33.8–61.4%	96.53%	
Sepsis	14	55.1%	44.6–65.6%	97.59%	
Mixed diagnoses	13	50.2%	38.4–62.0%	96.51%	
Surgery	3	43.0%	24.4–61.6%	0%	
**Muscle mass measurement**					0.01
CT	33	52.5%	45.5–59.4%	97.29%	
Anthropometric methods	2	36.9%	30.1–43.6%	0%	
Ultrasonography	2	40.1%	32.9–47.4%	0%	
**Sample size**					0.63
≥100	24	51.8%	43.5–60.2%	97.97%	
<100	13	49.2%	41.4–57.2%	84.47%	
**Age group**					0.79
≥60	21	51.5%	42.7–60.4%	97.0%	
<60	11	50.0%	38.9–61.0%	94.8%	

CT, computed tomography scan.

#### 3.5.2. Subgroup analysis by sex

Twenty-five studies provided the prevalence of LSMM by sex. The results showed no statistically significant difference in the prevalence of LSMM based on sex. The prevalence of LSMM was 48.8% (95% CI, 40.0–57.6%) among men and 45.5% (37.9–53.2%) among women (Table 2).

#### 3.5.3. Subgroup analysis by mechanical ventilation

Seventeen studies included critically ill patients who underwent mechanical ventilation during the hospitalization period. The results of this subgroup analysis showed that the prevalence of LSMM was slightly higher in patients with than without mechanical ventilation, at 53.4% (43.2–63.6%) and 48.9% (39.7–58.1%), respectively. However, there was no significant difference between these two groups (*P* = 0.44; Table 2).

#### 3.5.4. Subgroup analysis by diagnosis

We categorized the studies into four classifications based on the main diagnosis. Fourteen studies focused on sepsis, and the prevalence of LSMM among these participants was 55.1% (95% CI, 44.6–65.6%). Seven studies focused on critically ill patients with trauma, and the prevalence of LSMM was 47.6% (33.8–61.4%). Only three studies focused on surgical patients, among whom the prevalence of LSMM was 43.0% (24.4–61.6%). The remaining 13 studies involved patients with mixed diagnoses, and the pooled prevalence of LSMM was 50.2% (38.4–62.0%) (Table 2).

#### 3.5.5. Other subgroup analyses of prevalence of LSMM

We split the sample size into two groups (<100 vs. ≥100), and the prevalence of LSMM was similar between the two groups at 49.2% (41.4–57.2%) and 51.8% (43.5–60.2%), respectively. Additionally, a subgroup analysis between age groups split by 60 years showed no statistically significant difference between these two age groups (51.5%, 95%CI: 42.7–60.4%; vs. 50.0%, 95%CI: 38.9–61.0%) (Table 2).

### 3.6. Meta-analysis of association between LSMM and mortality risk

**Figure 3:**
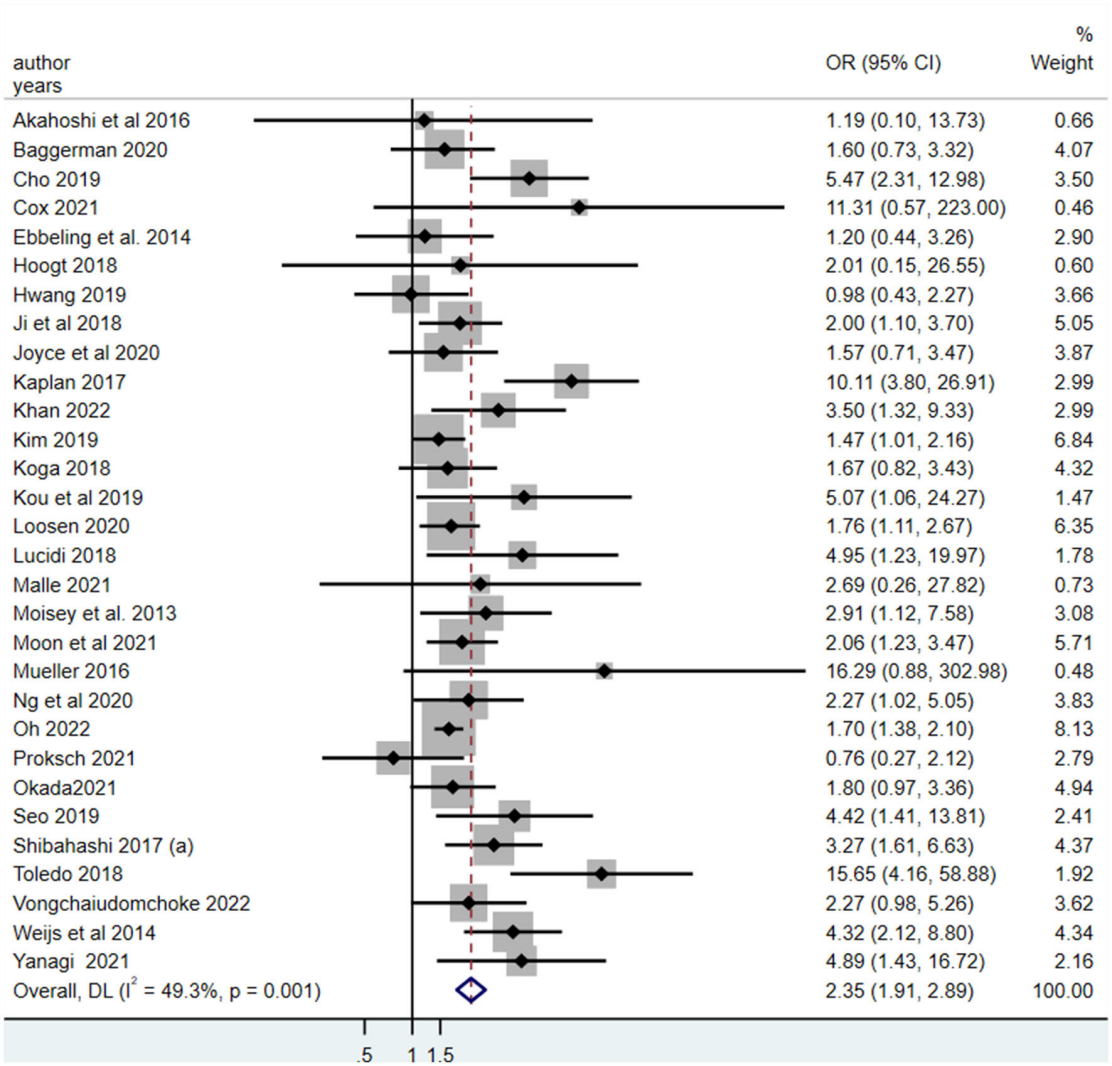
The meta-analysis for the association between the low skeletal muscle mass and all-cause mortality in critically ill patients.

Thirty studies explored the association between LSMM and mortality risk (19, 20, 24, 26–37, 39, 41–44, 46–48, 50–53, 55, 57, 58). The pooled odds ratio (OR) for the association between LSMM and mortality risk was 2.35 (95% CI, 1.91–2.89), which indicated that critically ill patients with LSMM had an approximately 2.35 higher likelihood of mortality than those without LSMM (Figure 3).

### 3.7. Subgroup analysis of association between LSMM and mortality risk by different variables

#### 3.7.1. Subgroup analysis based on mechanical ventilation

For critically ill patients who were mechanically ventilated, the pooled OR for the association between LSMM and mortality risk was 2.32 (95% CI, 1.85–2.90). Critically ill patients with LSMM who were not ventilated also had a high risk of mortality, with a pooled OR of 2.42 (95% CI, 1.74–3.36) (Table 3).

**Table 3:** Subgroup analyses of the association between low skeletal muscle mass and mortality in critically ill patients.

**Variables**	**Number of studies**	**OR**	**95%CI**	***I* ^2^**	***P*-value for difference**
**Country**					0.71
Asian	15	2.14	1.75–2.61	26.1%	
Europe	5	2.40	1.48–3.87	37.9%	
Americas	8	3.19	1.31–7.76	76.6%	
Oceania	2	1.66	0.78–3.52	0%	
**MV**					0.47
Yes	13	2.32	1.85–2.90	10.4%	
No	17	2.42	1.74–3.36	61.5%	
**Diagnose**					0.11
Trauma	5	2.14	0.79–5.83	74.0%	
Sepsis	12	1.80	1.56–2.09	0%	
Mixed diagnosis	11	3.21	2.01–5.13	56.9%	
Surgery	2	2.72	1.30–5.70	0%	
**Muscle mass measurement**					0.26
CT	26	2.25	1.82–2.80	51.4%	
Anthropometric methods	2	2.79	1.36–5.74	0%	
Ultrasonography	2	5.86	1.89–18.20	0%	
**Sample size**					0.21
≥100	22	1.95	1.72–2.21	54.7%	
<100	8	3.38	1.99–5.74	44.5%	
**Type of outcomes**					0.56
30-day mortality	6	3.23	1.54–6.75	56.4%	
In-hospital mortality	13	2.27	1.71–3.01	22.7%	
1-year mortality	6	2.60	1.67–4.06	77.4%	
Others	5	1.89	1.19–2.99	17.1%	

CT, computed tomography scan.

#### 3.7.2. Subgroup analysis based on outcomes

Critically ill patients with LSMM had a higher risk of mortality than those with normal skeletal muscle mass regardless of the type of outcome (in-hospital mortality, 30-day mortality, or 1-year mortality) with an OR of 2.27 (1.71–3.01), 3.23 (1.54–6.75), and 2.60 (1.67–4.06), respectively.

#### 3.7.3. Subgroup analysis based on assessment tools

We also performed a subgroup analysis based on the assessment tools used. The majority of the studies used CT for assessment of LSMM, and a few studies used anthropometric methods for defining LSMM; only two studies applied ultrasonography. The results showed that critically ill patients with LSMM had a higher mortality risk than those without LSMM when using CT or anthropometric methods; the pooled ORs were 2.25 (95% CI, 1.82–2.80) and 2.79 (1.36–5.74), respectively. We also found an independent association between LSMM and mortality risk among critically ill patients when using ultrasonography for confirmation (pooled OR,5.86; 95% CI, 1.89–18.20) (Table 3).

#### 3.7.4. Subgroup analysis based on diagnosis

Critically ill patients with LSMM had a higher mortality risk than those without LSMM among participants with sepsis (pooled OR, 1.80; 95% CI, 1.56–2.09), surgery (pooled OR, 2.72; 95% CI, 1.30–5.70), or mixed diagnosis (pooled OR, 3.21; 95% CI, 2.01–5.13). However, this association was not statistically significant among trauma patients (pooled OR, 2.14; 95% CI, 0.79–5.83) (Table 3).

#### 3.7.5. Other subgroup analyses of association between LSMM and mortality risk by country and sample size

We also performed subgroup analyses based on country and sample size. These results also showed that critically ill patients with LSMM had a higher mortality risk than their counterparts without LSMM, and the results were independent of country and sample size (Table 3).

### 3.8. Publication bias

We tested the publication bias for the outcomes of the prevalence of LSMM and mortality. The results showed no potential publication bias when pooling the prevalence of LSMM as indicated by Begg's test (*P*-value = 0.27) (Supplementary Figure 1a). However, the results of Begg's test (*P*-value = 0.05) showed potential publication bias when the results for the association between LSMM and mortality risk were pooled (Supplementary Figure 1b).

### 3.9. Quality assessment

The Newcastle–Ottawa Scale score ranged from 5 to 8 points, and most of the studies had 7 points. This result indicated that the quality of the included studies was relatively good (Supplementary Table 3).

### 3.10. The results of overall quality of evidence

The summary results of GRADE were showed in Supplementary Table 4, indicating that the evidence was low because there were few inconsistencies in some included studies and they were some publication bias across these studies.

### 3.11. Sensitivity analysis

A sensitivity analysis was performed by omitting one study and pooling the remaining studies to determine whether the pooled results showed major changes. The results of the sensitivity analysis regarding prevalence or mortality showed no significant changes (Supplementary Figure 2). In addition, we also conducted sensitivity analysis by omitting the studies that used anthropometric methods to define LSMM and the results was almost not changed, which indicated the results was stable (Supplementary Figure 3).

## 4. Discussion

Our study showed that the prevalence of LSMM among critically ill patients was very high at 51.0% (95% CI, 44.5–57.5%), meaning that more than half of critically ill patients had LSMM. This study also indicated that critically ill patients with LSMM had an approximately 2.35-fold higher mortality risk than those without LSMM. This systematic review and meta-analysis suggests that greater attention to LSMM, early screening of patients at high risk of LSMM, and timely interventions such as comprehensive treatments consisting of exercise and nutrition programs must be performed to slow down the process of muscle wasting. These efforts might reduce the mortality rate among critically ill patients.

Critically ill individuals often lose muscle mass for multiple reasons (17), such as extended time of inactivity, nutrient deficits, and impaired equilibrium between muscle protein synthesis and breakdown. In 2021, a systematic review of nine studies explored the association between LSMM defined by CT and mortality among critically ill patients (25). That study indicated that LSMM based on CT was associated with high short-term mortality. Notably, the study had two main limitations. First, their search strategy was relatively old studies, resulting in only 9 studies involving 1,563 patients, which might have influenced the representativeness of their findings. Second, the authors did not perform subgroup analyses based on different variables; such analyses are very important. Given that more researchers and clinicians are paying attention to LSMM in critically ill patients, increasing numbers of studies are focusing on this topic. Therefore, an updated meta-analysis that can provide an overall picture of the impact of LSMM on critical illness is required. Our study has overcome these limitations.

The pooled prevalence of LSMM in our study was very high at 51.0%. A previous systematic review showed that the pooled prevalence of LSMM was 46.2% (95% CI, 43.95–48.45%) among patients with metastasized colorectal cancer, which was very close to our results (59). We speculate that malnutrition, anorexia, and inflammatory reactions are common characteristics among patients with cancer and critically ill patients, leading to a high prevalence of LSMM (60). In addition, inactivity due to disease and treatment procedures places critically ill patients at high risk for LSMM. Therefore, early screening for LSMM among these select patients is very important.

The present study showed that critically ill patients undergoing mechanical ventilation might have a higher prevalence of LSMM than those without this treatment procedure, although there was no statistically significant difference. We speculate that LSMM may be worse in critically ill inpatients with than without mechanical ventilation, but this requires further study. Patients who require mechanical ventilation often have major lung disease. Mechanical ventilation prevents oral ingestion of food (61); instead, these patients require nasogastric tube feeding or other methods for nutrition and energy, which might not provide sufficient nutrition, thus reducing their protein intake. In addition, mechanical ventilation makes critically ill inpatients immobile. For these two main reasons, critical illness may be associated with a high prevalence of LSMM. Moreover, our study showed that the prevalence of LSMM among patients with sepsis was higher than that among patients with other diagnoses. The reason for this difference might be that patients with sepsis often have a high inflammatory response that leads to multiple organ disorders and malnutrition, resulting in a high risk of LSMM (62, 63). In addition, a previous study revealed that the mechanisms by which sepsis leads to LSMM are disorganized sarcomeres and myofibril dysfunction (64).

Our study also showed that patients with LSMM had a higher risk of mortality than those with normal muscle mass, and this result was in line with a previous study (25). LSMM is the main element in muscle wasting and has been widely confirmed to increase the risk of mortality across different populations (65–68). The mechanisms underlying the association between LSMM and mortality were elucidated in a previous study. In general, most critically ill patients with LSMM had serious comorbidities that increased their mortality risk. In addition, patients with LSMM may have decreased immune system function, which reduces their ability to resist the wide-ranging adverse effects of many treatment procedures such as mechanical ventilation, polypharmacy, and pulse index continuous cardiac output monitoring. Therefore, LSMM complicated by worsening of the patient's primary disease can become a vicious circle that results in a high likelihood of mortality.

Ultrasound measurement is a convenient, widely applied method with which to determine whether patients with critical illness have LSMM (69, 70). Multiple studies have compared the performance between B-mode ultrasound images and typical methods of defining LSMM (71–73). One study indicated that B-mode ultrasound imaging has great potential as a surrogate diagnostic tool for LSMM. Our study also showed a statistically significant association between LSMM and mortality, which is consistent with a previous study (74). One study in which ultrasound was used to detect muscle psoas indices showed that the psoas muscle index was associated with mortality, with a hazard ratio of 0.93 (95% CI, 0.876–0.987) (71). In addition, a study conducted in 2022 revealed that muscle thickness tested by ultrasound was independently associated with death among patients undergoing hemodialysis (73). Therefore, the association between LSMM based on ultrasound and mortality among critically ill patients should be further explored. Given the simplicity, practicality, and convenience of ultrasound for assessing muscle mass, we expect that this technique will provide prognostic value for predicting mortality among critically ill patients.

Our study had several strengths and limitations. First, compared with previous studies, this updated meta-analysis provided a more in-depth subgroup analysis of the pooled prevalence of LSMM and pooled the association between LSMM and mortality. The subgroup analysis based on diagnosis and measurement assessment for LSMM provides more valuable information that can guide clinical practice. Second, this meta-analysis included multiple studies involving 6,891 participants, thus providing a highly representative sample of this special population. Third, a comprehensive statistical analysis was used to test the robustness of our study. However, our meta-analysis also had some limitations. First, there was potential publication bias regarding the association between LSMM and mortality. Some non-English-language studies might have been excluded from the meta-analysis. Second, some studies used the results of a univariate analysis to assess the association between LSMM and mortality, potentially resulting in overestimation of the effect by confounding factors. Third, we considered the effect size by the hazard ratio to be equal to the OR in our study, and caution is therefore required when interpreting our main findings. Fourth, the overall quality of GRADE assessment was “low”, more large-scale and high-quality prospective cohort studies, especially those based on muscle ultrasound, are required to validate these findings.

## 5. Conclusion

Our study showed a high prevalence of LSMM among critically ill patients and revealed that critically ill patients with LSMM had an 2.35-fold higher mortality risk than those with normal muscle mass. This study indicates that the importance of muscle mass as a potentially important prognostic factor in critically ill patients. Healthcare providers were encouraged to carefully consider the risks and benefits of using CT for muscle mass measurement in critically ill patients, and to consider alternative methods such as ultrasound or bioelectrical impedance analysis (BIA) where appropriate. Early interventions, such as mobilizing patients and providing nutritional support, after confirming LSMM may reduce the mortality rate in this particular population.

## Data availability statement

The original contributions presented in the study are included in the article/Supplementary material, further inquiries can be directed to the corresponding authors.

## Author contributions

HY and X-XW were responsible for screening and extracting the studies. HM and ZL were responsible for study quality assessment. HY, LW, and X-MZ were responsible for statistical analysis. YX and X-MZ were responsible for the study design and review. HY, X-XW, and X-MZ drafted the manuscript. All authors contributed to the article and approved the submitted version.
