# Supplementary material for: Prevalence and mortality risk of low skeletal muscle mass in critically ill patients: an updated systematic review and meta-analysis
Source: Front Nutr. 2023 May 12;10:1117558. doi: 10.3389/fnut.2023.1117558 (PMC10213681; doi:10.3389/fnut.2023.1117558)
Supplement: Supplementary file 1 [file Data_Sheet_1.docx]

Supplementary File 1. Search Strategy

Search strategy

PubMed

#1 Search: (((((intensive care unit) OR intensive care) OR "Critical Care"[Mesh]) OR critically ill) OR critical illness) OR "Critical Illness"[Mesh] Sort by: Most Recent

#2 Search: skeletal muscle mass Sort by: Most Recent

#3 Search: (muscle wasting) Sort by: Most Recent

#4 Search: (low skeletal muscle) Sort by: Most Recent

#5 Search: ((skeletal muscle mass) OR ((muscle wasting))) OR ((low skeletal muscle)) Sort by: Most Recent

#6 Search: ((mortality) OR (death)) OR (survival) Sort by: Most Recent

#7 Search: ((((mortality) OR (death)) OR (survival)) AND (((skeletal muscle mass) OR ((muscle wasting))) OR ((low skeletal muscle)))) AND ((((((intensive care unit) OR intensive care) OR "Critical Care"[Mesh]) OR critically ill) OR critical illness) OR "Critical Illness"[Mesh]) Sort by: Most Recent

**Embase**

#1: ('muscle atrophy'/exp OR 'amyotrophia' OR 'amyotrophy' OR 'atrophic muscular disorders' OR 'atrophy type 2' OR 'atrophy, muscle' OR 'degeneration, muscle' OR 'hirayama disease' OR 'muscle atrophia' OR 'muscle atrophy' OR 'muscle cell degeneration' OR 'muscle degeneration' OR 'muscle fiber atrophy' OR 'muscle fiber degeneration' OR 'muscle recession' OR 'muscle wasting' OR 'muscular atrophy' OR 'muscular degeneration' OR 'muscular disorders, atrophic' OR 'myoatrophy' OR 'myodegeneration' OR 'myofibrillar degeneration' OR 'myophagism' OR 'skeletal muscle mass'/exp OR 'low skeletal muscle') AND ('mortality'/exp OR 'mortality' OR 'mortality model' OR 'death'/exp OR 'survival'/exp) AND ('critical illness'/exp OR 'critically ill patient'/exp OR 'critically ill' OR 'critically ill patient' OR 'intensive care unit'/exp)

#2: ('case control study'/de OR 'clinical article'/de OR 'clinical study'/de OR 'clinical trial'/de OR 'clinical trial topic'/de OR 'cohort analysis'/de OR 'comparative study'/de OR 'controlled clinical trial'/de OR 'cross sectional study'/de OR 'human'/de OR 'intervention study'/de OR 'longitudinal study'/de OR 'major clinical study'/de OR 'multicenter study'/de OR 'observational study'/de OR 'outcomes research'/de OR 'prospective study'/de OR 'quality control'/de OR 'retrospective study'/de)

#3 : #1 AND #2
