# Supplementary material for: Prevalence and mortality risk of low skeletal muscle mass in critically ill patients: an updated systematic review and meta-analysis
Source: Front Nutr. 2023 May 12;10:1117558. doi: 10.3389/fnut.2023.1117558 (PMC10213681; doi:10.3389/fnut.2023.1117558)
Supplement: Supplementary file 3 [file Table_1.docx]

**Supplementary Table 1: The time for assessing muscle mass**

| Number | Name | Time of assessing muscle |
| --- | --- | --- |
| 1 | Akahoshi 2016 | Within 1 h of patient arrival by ambulance |
| 2 | Baggerman 2020 | Between 6 days before and 2 days after ICU admission |
| 3 | Baretto 2019 | Within 28-days of the creatinine and cystatin C concentrations |
| 4 | Cho 2019 | Before ICU admission |
| 5 | Cox 2021 | Within 48 h of sepsis diagnosis |
| 6 | Damanti 2021 | During hospitalization |
| 7 | Ebbeling 2014 | On ICU admission |
| 8 | Hoogt 2018 | Between 3 months before admission |
| 9 | Hwang 2019 | On ICU admission |
| 10 | Ji 2018 | Before preoperative period |
| 11 | Joyce 2020 | Between 7 days prior to and 1 day after admission to ICU |
| 12 | Ju 2020 | Before admission to the MICU |
| 13 | Kaplan 2017 | Within 48 hours of admission to ICU |
| 14 | Khan 2022 | Before the ICU admission |
| 15 | Kim 2019 | Within 24 h of ICU admission |
| 16 | koga 2018 | Within 24 h of ICU admission |
| 17 | Kou 2019 | Within 30 days of the first Spontaneous breathing trial |
| 18 | Looijaard 2020 | During ICU admission |
| 19 | Loosen 2020 | On ICU admission |
| 20 | Lucidi 2018 | At admission |
| 21 | Malle 2021 | 6 months before and 3 days after ICU admission |
| 22 | Moisey 2013 | On the day of admission |
| 23 | Moon 2021 | Two days before ICU admission |
| 24 | Mueller 2016 | Before ICU admission |
| 25 | Ng 2020 | Within 72 hours after ICU admission |
| 26 | Oh 2022 | Within 24 h of hospitalization |
| 27 | Okada 2021 | Within 24 h of hospital arrival |
| 28 | Proksch 2021 | On admission or within previous 3 months |
| 29 | Seo 2019 | CT image of 90–180 days before septic shock diagnosis |
| 30 | Sheean 2014 | On the 14 days of admission |
| 31 | Shibahashi 2017(a) | On the day of admission |
| 32 | Shibahashi 2017(b) | On the day of admission |
| 33 | Toledo 2018 | At ICU admission |
| 34 | Vongchaiudomchoke 2022 | Within 24 h of enrollment |
| 35 | Weijs 2014 | between 1 day before and 4 days after ICU admission |
| 36 | Woo 2020 | Within 3 days before or after intubation |
| 37 | Xi 2021 | Within 1 week of admission |
| 38 | Yanagi 2021 | Within 1 week of ICU admission |
