# Supplementary material for: Prevalence and mortality risk of low skeletal muscle mass in critically ill patients: an updated systematic review and meta-analysis
Source: Front Nutr. 2023 May 12;10:1117558. doi: 10.3389/fnut.2023.1117558 (PMC10213681; doi:10.3389/fnut.2023.1117558)
Supplement: Supplementary file 4 [file Table_2.docx]

**Supplemental Table 2: The details of diagnosis criteria and cut-off points of each study**

| Number | Name | Cut-off Values | Part of body |
| --- | --- | --- | --- |
| 1 | Akahoshi 2016 | SMA < 80 % estimated SMA for both sexes | L3 caudal end |
| 2 | Baggerman 2020 | Males: SMI< 41.6 cm2/m2  Females: SMI< and 32.0 cm2/m2 | L3 |
| 3 | Baretto 2019 | Male: SMI <43 cm2/m2  Female: SMI <41 cm2/m2 | L3 |
| 4 | Cho 2019 | Male: SMI < 5.45cm2/m2  Female: SMI < 3.85cm2/m2 | L3 |
| 5 | Cox 2021 | Male: SMI <=52.4 cm2/m2  Female: SMI <=38.5 cm2/m2 | L3 |
| 6 | Damanti 2021 | Females: Skeletal muscle area (SMA) <92.2 cm2,  Males: SMA <144.3 cm2 | L3 |
| 7 | Ebbeling 2014 | Vertebral Index < 50 percentile of PLVI (≤ 0.83) for both sexes | Psoas L4 |
| 8 | Hoogt 2018 | Not available | L3 |
| 9 | Hwang 2019 | Males :SMI < 55.4 cm2/m2  Females: SMI < 38.9 cm2/m2 | L3 |
| 10 | Ji 2018 | Male: SMI ≤ 40.8 cm^2^ /m^2^ ; and Female: SMI≤ 34.9 cm^2^ /m^2^ | L3 |
| 11 | Joyce 2020 | Females: SMA < 110 cm^2^ ; and male: SMA< 170 cm^2^ | L3 |
| 12 | Ju 2020 | Females: SMI ≤ 38.5 cm2/m2; male ≤ 52.4 cm2/m2 | L3 |
| 13 | Kaplan 2017 | Males: SMI < 52.4 cm2/m2  Female: SMI< 38.5cm2/m2 | L3 |
| 14 | Khan 2022 | Male: SMI <50 cm/m2 ; Female: SMI <39 cm/m2 | L3 |
| 15 | Kim 2019 | Males: SMI <55 cm2/m2 for  Females: SMI <39 cm2/m2 for females | L3 |
| 16 | koga 2018 | SMA < 80 % estimated SMA for both sexes | L3 |
| 17 | Kou 2019 | Females: TPA <385 mm^2^ /m^2^ ;  Male: TPA<545 mm^2^ /m^2^ | L3 |
| 18 | Looijaard 2020 | Males: SMA <170 cm2  Females: SMA <110 cm2 | L3 |
| 19 | Loosen2013 | Skeletal muscle index (L3SMI) < 74.95mm2/cm for both sexes | L3 |
| 20 | Lucidi 2018 | Mid-arm muscle circumference (MAMC) <5th percentile for both sexes | Mid-arm muscle circumference |
| 21 | Malle 2021 | Female: TPA <642mm2/m2  Male: TPA< 784mm2/m2 | L3 |
| 22 | Moisey 2013 | Females: SMI≤ 38.9 cm^2^ /m^2^  Males: SMI≤ 55.4 cm^2^ /m^2^ for males | L3 |
| 23 | Moon 2021 | Cross-sectional area (CSA) values below the median value | Fourth thoracic vertebral region |
| 24 | Mueller 2016 | RFCSA ≤ 5.2 cm2 for both sexes | Rectus femoris cross-sectional area |
| 25 | Ng 2020 | SMI < 42.0 cm2/m2 for both sexes | L3 |
| 26 | Oh 2022 | SMI <45.4 cm2/m2 in  men and 34.4 cm2/m2 in women | L3 |
| 27 | Okada 2021 | Psoas index≤ 0.68 for both sexes | L3 |
| 28 | Proksch 2021 | Not available | L4 vertebra |
| 29 | Seo 2019 | Total Abdominal Muscle Area Index  Female: low TAMAI was defined as <41 cm2/m2  Male: low TAMAI <43 cm2/m2 when BMI was <25 kg/m2,  and <53 cm2/m2 when BMI was 25 kg/m2 or more. | L3 |
| 30 | Sheean 2014 | Female: SMI ≤ 38.5 cm^2^ /m^2^  Male SMI≤ 52.4 cm^2^ /m^2^ | L3 |
| 31 | Shibahashi 2017(a) | Male: SMA < 45.2 cm2  Female: SMA < 39.0 cm2 | L3 |
| 32 | Shibahashi 2017(b) | Male: SMA < 58.9 cm2  Female SMA < 39.0 cm2 | L3 |
| 33 | Toledo 2018 | Male: SMI < 55.27 cm2/m2  Female: SMI < 40.13 cm2/m2 | L3 |
| 34 | Vongchaiudomchoke 2022 | low muscle mass (for male, a muscle mass/bodyweight2 < 7.0 kg/m2; and for female, < 5.7 kg/m2) | BIA |
| 35 | Weijs 2014 | Female: SMA < 110 cm^2^  Male: SMA <170 cm^2^ for males | L3 |
| 36 | Woo 2020 | Male: SMI ≤ 49 cm^2^ /m^2^  Female: SMI ≤ 31 cm^2^ /m^2^ | L3 |
| 37 | Xi 2021 | Male: SMI≤ 42.08 cm^2^ /m^2^  Female: SMI≤ 37.35 cm^2^ /m^2^ | L3 |
| 38 | Yanagi 2021 | muscle thickness < 20.9 mm for both sexes | Quadriceps femoris muscle |
