# Supplementary material for: Prevalence and mortality risk of low skeletal muscle mass in critically ill patients: an updated systematic review and meta-analysis
Source: Front Nutr. 2023 May 12;10:1117558. doi: 10.3389/fnut.2023.1117558 (PMC10213681; doi:10.3389/fnut.2023.1117558)
Supplement: Supplementary file 5 [file Table_3.docx]

**Supplementary Table 3: The results of Newcastle-Ottawa scale**

| Newcastle-Ottawa scale | Selection (1) |  |  |  | Comparability (2) | Outcome (3) |  |  | Total |
| --- | --- | --- | --- | --- | --- | --- | --- | --- | --- |
|  | Representativeness  of the exposed cohort | Selection of the non-exposed cohort | Ascertainment of exposure | Outcomes were not present at study initiation | Comparability of cohorts on the basis of the design or analysis | Assessment of outcome | Was follow-up long enough for outcome to occur | Adequacy  of follow-up |  |
| Akahoshi 2016 | 1 | 1 | 1 | 1 | 1 | 1 | 0 | 1 | 7 |
| Baggerman 2020 | 1 | 1 | 1 | 1 | 1 | 1 | 1 | 0 | 7 |
| Baretto 2019 | 1 | 1 | 1 | 1 | 1 | 1 | 1 | 0 | 7 |
| Cho 2019 | 1 | 1 | 1 | 1 | 1 | 1 | 1 | 1 | 8 |
| Cox 2021 | 0 | 1 | 1 | 1 | 2 | 1 | 1 | 1 | 8 |
| Damanti 2021 | 1 | 1 | 1 | 1 | 1 | 1 | 0 | 0 | 6 |
| Ebbeling 2014 | 1 | 1 | 1 | 1 | 1 | 1 | 1 | 0 | 7 |
| Hoogt 2018 | 1 | 1 | 1 | 1 | 1 | 1 | 0 | 0 | 6 |
| Hwang 2019 | 1 | 1 | 1 | 1 | 1 | 1 | 0 | 0 | 6 |
| Ji. 2018 | 1 | 1 | 1 | 1 | 1 | 1 | 1 | 1 | 8 |
| Joyce 2020 | 0 | 1 | 1 | 1 | 1 | 1 | 1 | 1 | 7 |
| Ju 2020 | 1 | 1 | 1 | 1 | 1 | 1 | 0 | 0 | 6 |
| Kaplan 2017 | 1 | 1 | 1 | 1 | 1 | 1 | 1 | 1 | 8 |
| Khan 2022 | 1 | 1 | 1 | 1 | 1 | 1 | 0 | 0 | 6 |
| Kim 2019 | 1 | 1 | 1 | 1 | 1 | 1 | 0 | 1 | 7 |
| Koga 2018 | 1 | 1 | 1 | 1 | 1 | 1 | 0 | 0 | 6 |
| Kou 2019 | 1 | 1 | 1 | 1 | 1 | 1 | 0 | 0 | 6 |
| Looijaard 2020 | 1 | 1 | 1 | 1 | 1 | 1 | 0 | 0 | 6 |
| Loosen2020 | 1 | 1 | 1 | 1 | 1 | 1 | 0 | 1 | 7 |
| Lucidi 2018 | 0 | 1 | 1 | 1 | 1 | 1 | 0 | 0 | 5 |
| Malle 2021 | 0 | 1 | 1 | 1 | 1 | 1 | 0 | 1 | 6 |
| Moisey 2013 | 1 | 1 | 1 | 1 | 2 | 1 | 0 | 0 | 7 |
| Moon 2021 | 1 | 1 | 1 | 1 | 1 | 1 | 0 | 0 | 6 |
| Mueller 2016 | 1 | 1 | 1 | 1 | 1 | 1 | 0 | 0 | 6 |
| Ng 2020 | 1 | 1 | 1 | 1 | 1 | 1 | 1 | 0 | 7 |
| Oh 2022 | 1 | 1 | 1 | 1 | 2 | 1 | 0 | 1 | 8 |
| Okada 2021 | 1 | 1 | 1 | 1 | 2 | 1 | 0 | 1 | 8 |
| Proksch 2021 | 1 | 1 | 1 | 1 | 0 | 1 | 0 | 1 | 6 |
| Seo 2019 | 1 | 1 | 1 | 1 | 1 | 1 | 0 | 1 | 7 |
| Sheean 2014 | 1 | 1 | 1 | 1 | 0 | 1 | 0 | 0 | 5 |
| Shibahashi 2017 (a) | 1 | 1 | 1 | 1 | 0 | 1 | 0 | 0 | 5 |
| Shibahashi 2017(b) | 1 | 1 | 1 | 1 | 0 | 1 | 0 | 0 | 5 |
| Toledo 2018 | 1 | 1 | 1 | 1 | 1 | 1 | 0 | 1 | 7 |
| Vongchaiudomchoke 2022 | 1 | 1 | 1 | 1 | 1 | 1 | 1 | 1 | 8 |
| Weijs 2014 | 1 | 1 | 1 | 1 | 1 | 1 | 0 | 0 | 6 |
| Woo 2020 | 1 | 1 | 1 | 1 | 1 | 1 | 0 | 0 | 6 |
| Xi 2021 | 1 | 1 | 1 | 1 | 1 | 1 | 0 | 0 | 6 |
| Yanagi 2021 | 1 | 1 | 1 | 1 | 0 | 1 | 0 | 1 | 6 |
