# Supplementary material for: Prevalence and mortality risk of low skeletal muscle mass in critically ill patients: an updated systematic review and meta-analysis
Source: Front Nutr. 2023 May 12;10:1117558. doi: 10.3389/fnut.2023.1117558 (PMC10213681; doi:10.3389/fnut.2023.1117558)
Supplement: Supplementary file 6 [file Table_4.docx]

**Supplemental Table 4: Overall evidence quality by Grade**

| **Quality assessment** | | | | | | | | **Quality importance** | | |
| --- | --- | --- | --- | --- | --- | --- | --- | --- | --- | --- |
| **Outcome** | **Number of studies** | **Design** | **Risk of bias** | **Inconsistency** | **Indirectness** | **Imprecision** | **Other considerations** | **Relative (95% CI)** |  |  |
| All-cause mortality | 30 | observational studies | Serious | Serious | no serious indirectness | no serious imprecision | strong  association | OR= 2.35 (1.91 to 2.89) | ÅÅOO LOW | CRITICAL |
