# Supplementary figures and images for: Prevalence and mortality risk of low skeletal muscle mass in critically ill patients: an updated systematic review and meta-analysis
Source: Front Nutr. 2023 May 12;10:1117558. doi: 10.3389/fnut.2023.1117558 (PMC10213681; doi:10.3389/fnut.2023.1117558)

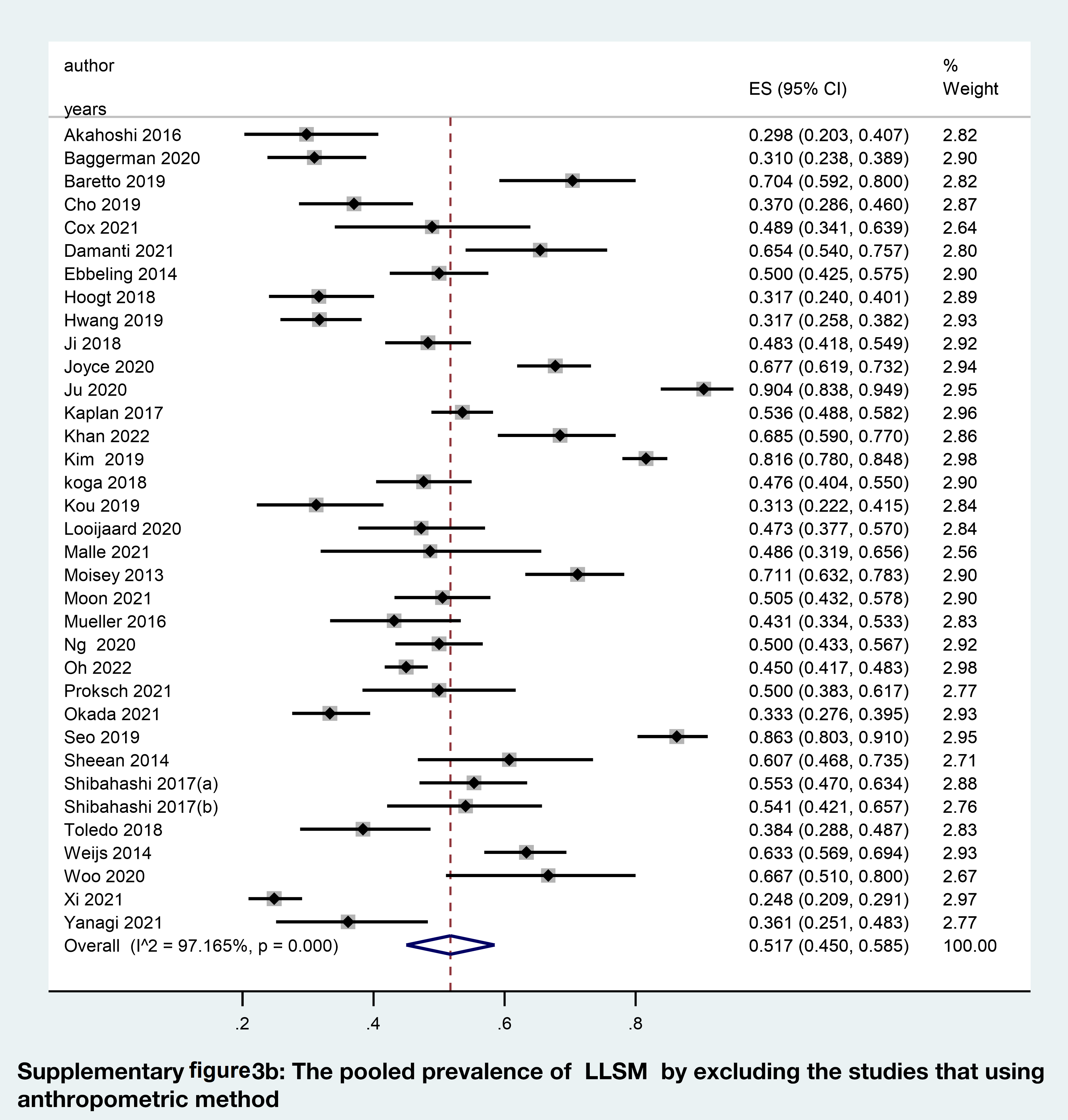

Supplement: Supplementary file 7 [file Data_Sheet_3.zip › Supplementary_Figure3b.TIF]

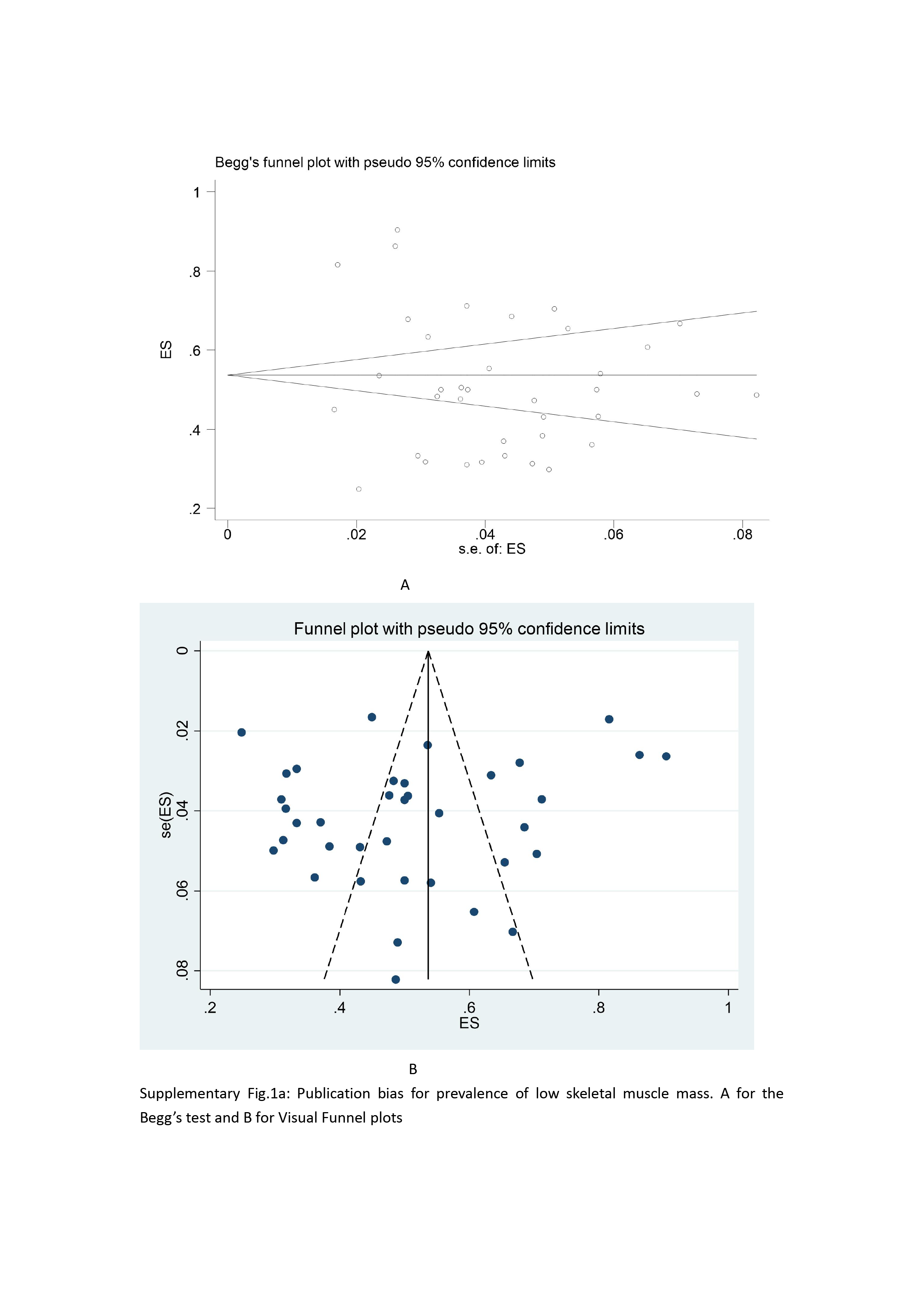

Supplement: Supplementary file 7 [file Data_Sheet_3.zip › Supplementary_Figure1a.TIF]

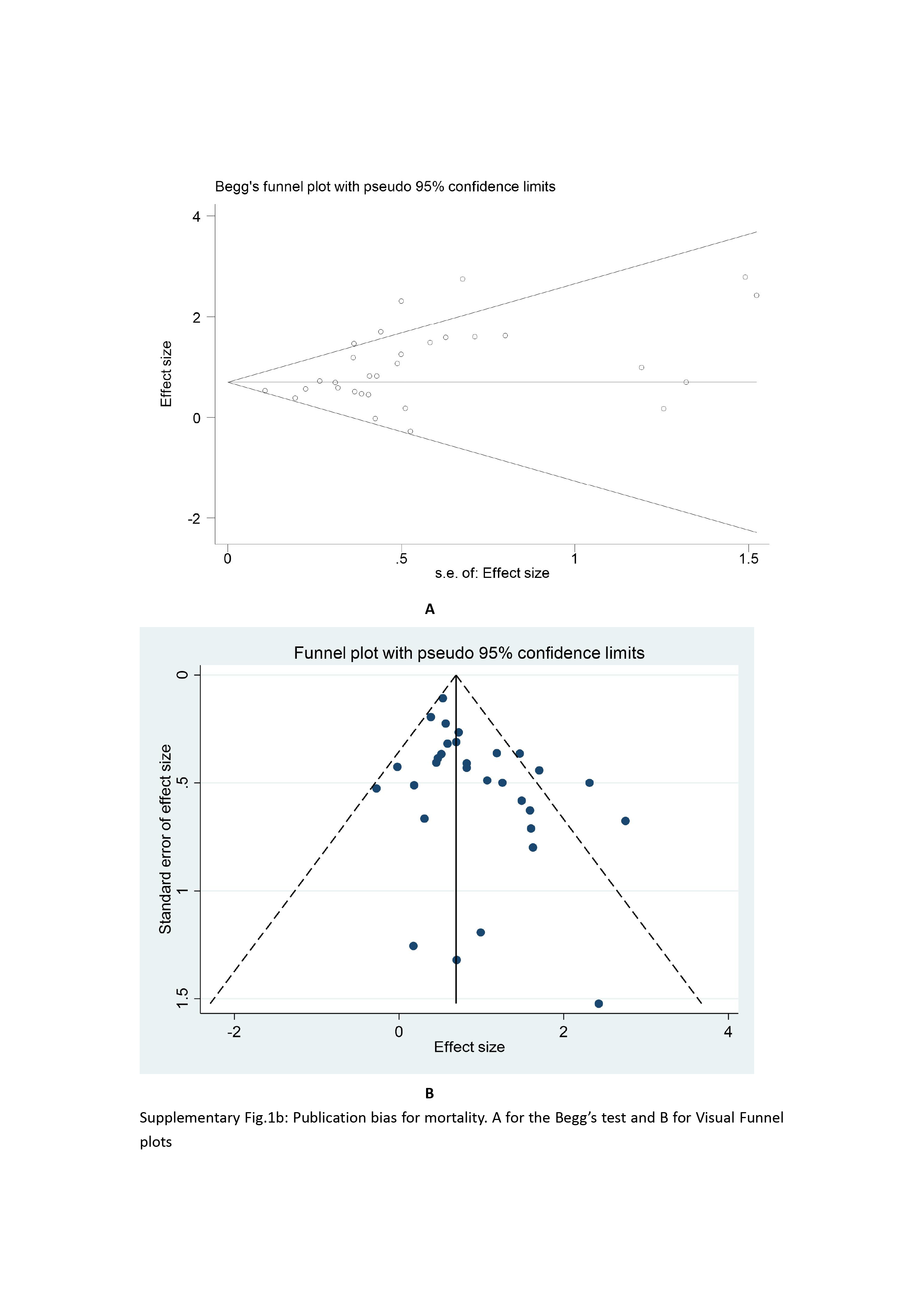

Supplement: Supplementary file 7 [file Data_Sheet_3.zip › Supplementary_Figure1b.TIF]

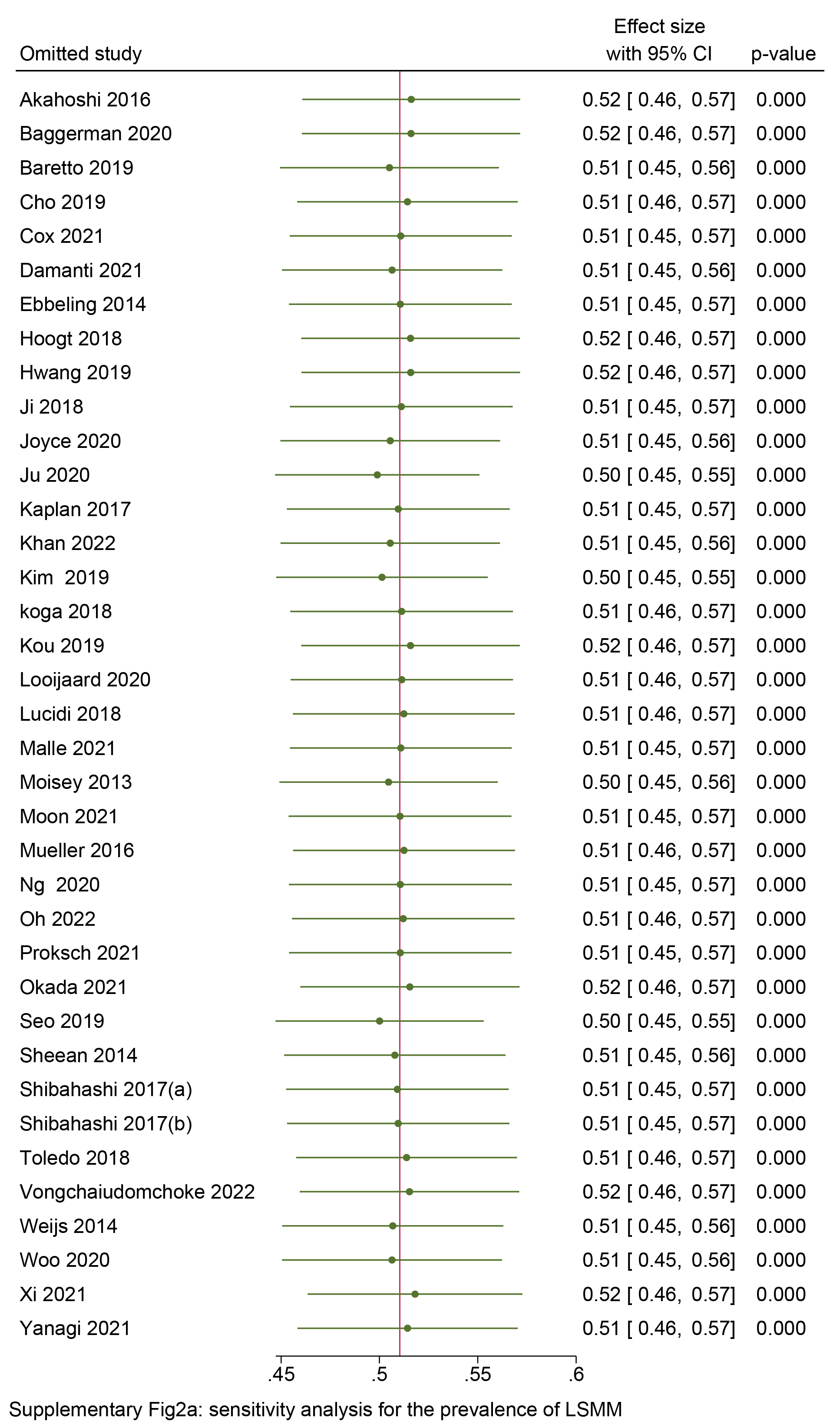

Supplement: Supplementary file 7 [file Data_Sheet_3.zip › Supplementary_Figure2a.TIF]

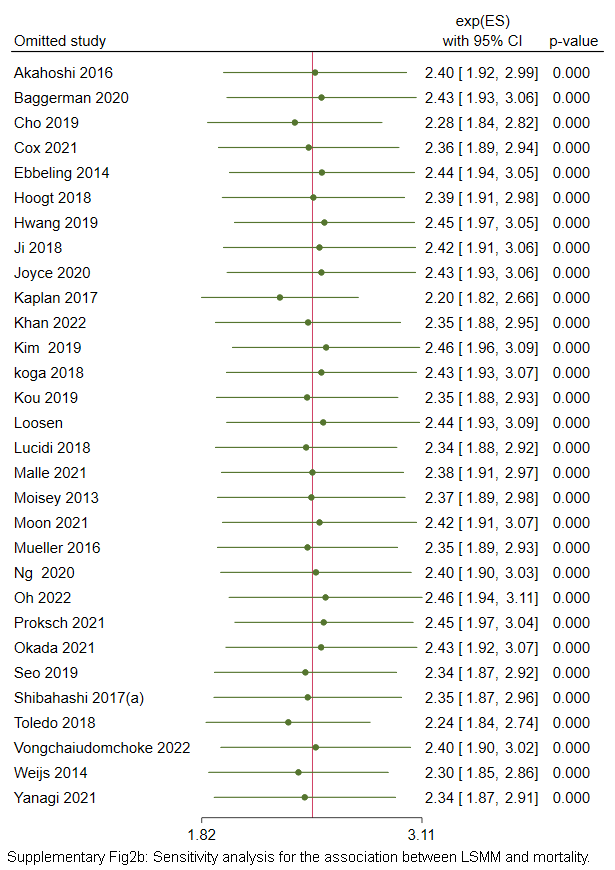

Supplement: Supplementary file 7 [file Data_Sheet_3.zip › Supplementary_Figure2b.TIF]

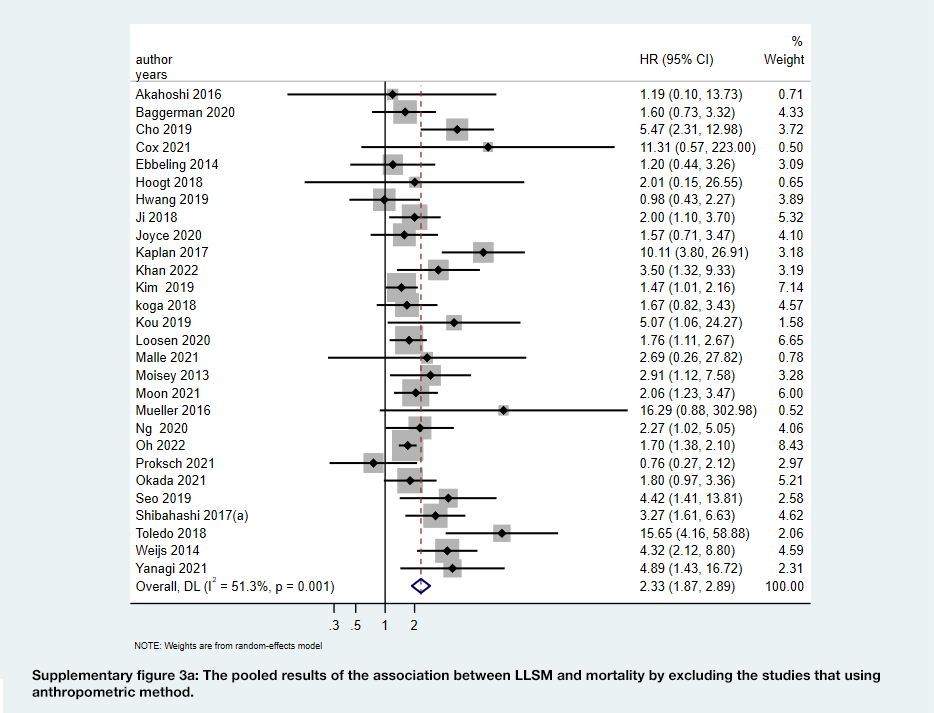

Supplement: Supplementary file 7 [file Data_Sheet_3.zip › Supplementary_Figure3a.TIF]
